# Supplementary material for: Mesenchymal Stem Cell-Mediated Deep Tumor Delivery of Gold Nanorod for Photothermal Therapy
Source: Nanomaterials (Basel). 2022 Sep 28;12(19):3410. doi: 10.3390/nano12193410 (PMC9565344; doi:10.3390/nano12193410)
Supplement: Supplementary file 1 [file nanomaterials-12-03410-s001.zip › nanomaterials-1949719-supplementary.pdf]

nanomaterials MDPI

Supplementary Material

Mesenchymal stem cell-mediated deep tumor delivery of gold nanorod for photothermal therapy

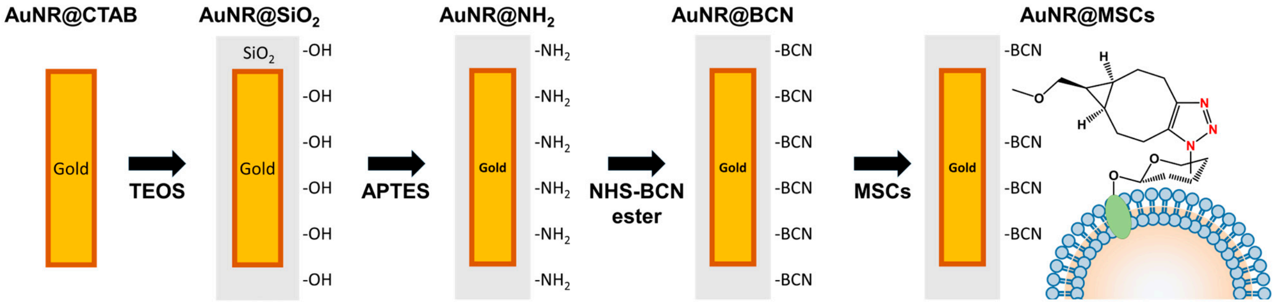

Figure S1. Schematic illustration to show the protocol for preparation of AuNR@MSCs.

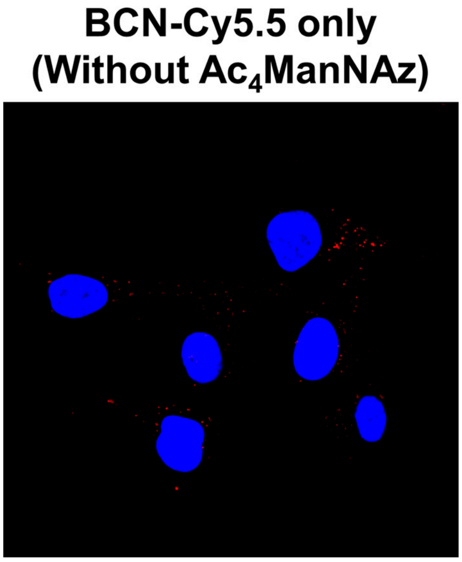

Figure S2. Fluorescence image of MSCs treated with BCN-Cy5.5 (without $Ac_4ManNAz$ treatment) for 48 h.

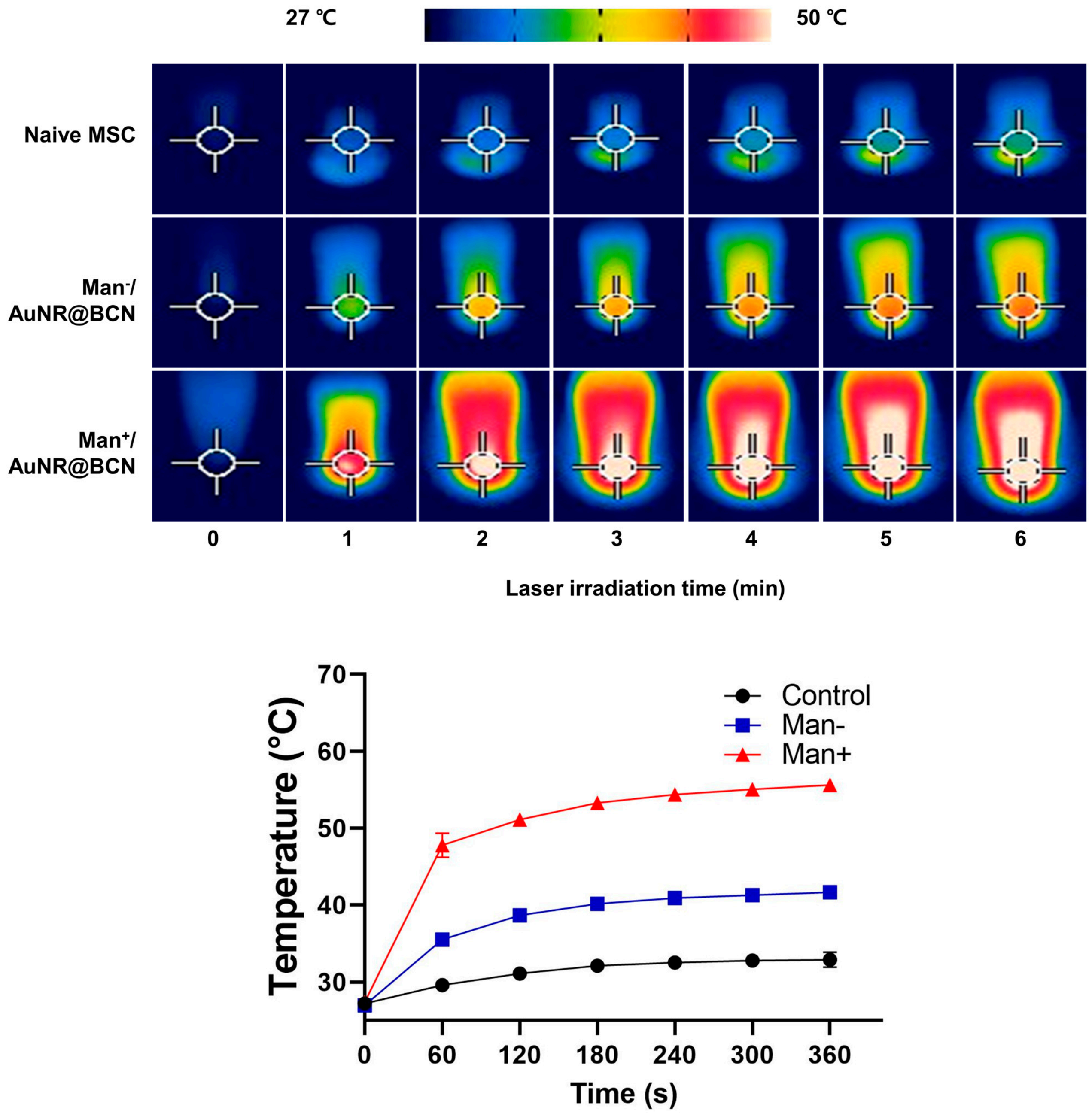

Figure S3. The photothermal efficiency of Man^{+}/AuNR@BCN and Man^{-}/AuNR@BCN under light irradiation with power of 1.0 W/cm^{2}.
